# Utilizing Extracorporeal Shockwave Therapy for in-Season Athletes

**DOI:** 10.3390/healthcare11071006

**Published:** 2023-04-01

**Authors:** Hye Chang Rhim, Joanne Borg-Stein, Steven Sampson, Adam S. Tenforde

**Affiliations:** 1 Department of Physical Medicine and Rehabilitation, Harvard Medical School, Boston, MA 02115, USA; hrhim@mgh.harvard.edu (H.C.R.);; 2 David Geffen School of Medicine, University of California, Los Angeles, CA 90095, USA

## Introduction

An athlete’s health and availability to train and compete at an optimal performance level is a growing focus for professional sports organizations. Back-to-back competitions with limited recovery time, along with travelling across multiple time zones, are inherent challenges [1]. Studies suggest that a higher rate of injuries are sustained during the pre-season and during competitions [2]. These injuries include, but are not limited to, muscle strains [3], tendinopathies [4], and injuries to bone [1,5]. Athletic injuries can be challenging to treat in-season, with unpredictable healing times following treatment interventions. Surgical management is typically reserved for off-season athletes, as recovery commonly requires a substantial rehabilitation period of 6 to 9 months post-operatively [4]. Identifying methods to treat injuries that exert positive clinical effects within 3 months would be desirable for in-season athletes. Emerging research suggests that extracorporeal shockwave therapy (ESWT) may represent an effective treatment to address sports-related injuries for in-season athletes and accelerate return to play.

## What Is ESWT?

Shockwaves are a type of energy that has biological effects at cellular, tissue, and organ levels. Some of the proposed mechanisms of action for ESWT include increased collagen synthesis [6], cellular proliferation and wound healing [7,8], pain reduction [9], and neovascularization [10]. ESWT currently has two primary modes of delivery: radial shockwave therapy (R-SWT) and focused shockwave therapy (F-SWT). R-SWT generates pressure waves that reach lower speeds and have lower peak pressure and therefore work on more superficial structures. On the other hand, F-SWT has the capacity to achieve deeper penetration from the site of application [11]. While both forms of ESWT have been used to treat a variety of conditions, the differences in their mechanistic effects may lead to different outcomes for a given condition.

ESWT has been shown to be effective in common athletic injuries, including plantar fasciitis [12], Achilles tendinopathy [13], medial tibial stress syndrome [14], and proximal hamstring tendinopathy [15]. Some of the potential side effects include post-procedural pain, skin erythema, skin bruising, hematoma formation, nerve irritation, and superficial edema [11]. ESWT has been known to be effective for the longitudinal management of musculoskeletal injuries and has a favorable safety profile, with recent work suggesting feasibility to support utilization of treatment in the care of in-season athletes.

## What High-Level Evidence Is Available to Support the Use of ESWT for In-Season Athletes?

Muscle injury: ESWT was shown to increase muscle elasticity, muscular tone, and muscular recruitment in selected muscles of healthy athletes within 30 days after 3 sessions of ESWT [16], suggesting its potential role in muscle recovery. A single session of F-SWT was found to provide relief of pain, increase in force, and improve pain-associated impairments to daily living following eccentric exercise-induced, delayed-onset muscle soreness [17].

Chronic injuries: Return to sport may be accelerated by the use of ESWT with medial tibial stress syndrome and bone trauma.

A prospective observational study evaluated athletes treated with a graded running program and F-SWT over 9 weeks for medial tibial stress syndrome. The authors demonstrated significantly faster time to full recovery, defined by running 18 min consecutively without pain, compared to a graded running program alone [18]. A randomized controlled trial in military cadets with medial tibial stress syndrome observed one session of F-SWT and exercise program resulted in longer running capacity and improvement in pain at four weeks [19].

A randomized controlled trial investigated European football players with groin pain with bone edema of the pubic bone (osteitis pubis). Visual analog scale for pain and Hip Disability and Osteoarthritis Outcome Score were significantly improved in the group receiving ESWT at 1 and 3 months. The athletes who received ESWT were also able to return to football significantly earlier (73.2 days vs. 102.6 days) [20].

One randomized controlled trial in jumping athletes identified limited relief at one week and no sustained benefit of 3 sessions of F-SWT for management of patellar tendinopathy during the competitive season [21]. The authors attributed these findings possibly to not adding other exercises or treatments such as eccentric training and not modifying training or competitions.

## What Are the Advantages of ESWT over Alternative Injection Treatments Such as Corticosteroids and Platelet-Rich Plasma?

Corticosteroid injections may offer rapid relief, but may increase the risk of atrophy, pain, and tendon or soft tissue rupture [22]. Furthermore, needle injection therapies in an athlete have potential complications, including infection, bleeding, increased pain, and prolonged recovery time [22,23]. Unlike these interventions, athletes may continue activities as tolerated with ESWT [24]. R-SWT for acute muscle injuries was tolerated well by elite European football players, even when applied on a daily basis [25]. Furthermore, while F-SWT alone was not shown to be beneficial in jumping athletes, the athletes continued to participate in both training and matches without noticeable adverse events during treatment [21].

Platelet-rich plasma is commonly used for sports medicine injuries given the mechanism of action to stimulate tissue healing, reduce pain, without concerns of cellular toxicity [26]. A meta-analysis, including five randomized controlled trials, suggests that the use of platelet-rich plasma may result in an earlier return to sport in the treatment of acute grade I or II muscle strains, excluding those isolated to the hamstring muscle [27]. Despite this finding, based on the systematic review of post-procedure protocols following platelet-rich plasma injections, weight bearing limitation or activity limitation has been imposed immediately after the injection of platelet-rich plasma and up to 7 days and return to play restricted up to 4-6 weeks [28]. A similar lack of clinical results is observed in the management of acute hamstring injuries in competitive and recreational athletes with intramuscular platelet-rich plasma [29]. However, this involved substandard platelet concentration and dosage, limiting researchers to the conclusion that plasma concentrates rather than platelet-rich plasma is ineffective for acute hamstring injuries.

## Clinical Applications and Future Work

R-SWT is classified as a low-risk device with a good safety profile that can be used for in-season athletes to facilitate their return to sport in selected injuries. The use of ESWT may be favorable for in-season athletes following load management strategies to allow for healing, or as a bridge for more advanced procedures such as platelet-rich plasma or surgery for off-season use.

There are some case series and reports that support the use of ESWT on physically active or athletic populations with early outcomes for sports injuries including acute muscle injuries [25], myositis ossificans [30,31], plantar fasciitis [32], Achilles tendinosis [33], proximal hamstring tendinopathy [33], and non-union stress fractures [24,34]. Future high-quality studies should aim to capture larger cohorts of athletes as well as early outcomes such as 3 and 6 weeks, not limited to outcomes 12 weeks or beyond that have been commonly reported. ESWT should also be considered an adjunctive tool for managing commonly encountered athletic injuries. Future research will explore its combination with orthobiologics to help with perioperative and post-operative recovery, including anterior cruciate ligament reconstruction [35].

## Conclusions

ESWT offers the potential to accelerate recovery from athletic injury and save athletic clubs valuable resources. Further human athlete studies using ESWT on larger cohorts are needed to explore optimal indications, dosing, and frequency parameters.

## Data Availability

Not applicable.
